# EXPERIENCES OF DISTRICT NURSES WITH MAKING DECISIONS ABOUT THE USAGE OF INVOLUNTARY TREATMENT

**DOI:** 10.1093/geroni/igac059.2383

**Published:** 2022-12-20

**Authors:** Vincent Moermans, Michel Bleijlevens, Hilde Verbeek, Bernadette Dierckx de Casterlé, Koen Milisen, Jan Hamers

**Affiliations:** Maastricht University, Maastricht, Limburg, Netherlands; Maastricht University, Maastricht, Limburg, Netherlands; Maastricht University, Maastricht, Limburg, Netherlands; KU Leuven, Leuven, Vlaams-Brabant, Belgium; KU Leuven, Leuven, Vlaams-Brabant, Belgium; Maastricht University, Maastricht, Limburg, Netherlands

## Abstract

District nurses play a key role in care of older Persons Living with Dementia (PLWD) who still live in their own home environment. Studies show that they often have to deal with decision-making about and the usage of involuntary treatment. Involuntary treatment is care provided that they resist to and/or have not given consent to, such physical restraints, psychotropic medication. This study aimed to describe district nurses’ experiences regarding the decision-making processes about and the usage of involuntary treatment among PLWD. A qualitative study design with a grounded theory approach was used. During semi-structured interviews, 16 district nurses shared experiences in 31 different cases of involuntary treatment use among PLWD. Results show that district nurses were often not involved in the decision-making process. In most cases, family caregivers decided solely to use involuntary treatment, and district nurses were asked to do also. District nurses experienced these situations as difficult to deal with. They felt that they were torn between providing “good care”, wishes of PLWD, and meeting the expectations of family caregivers. Their experiences were negatively affected if PLWD verbally rejected the care provided, they were unable to enter into a dialogue with the caregivers about the care provided, and if they did not have sufficient knowledge about how to deal with PLWD. These findings indicate the importance, of proactive case management in dementia care at home. District nurses can facilitate this by going into dialogue between PLWD and his caregivers, in order to provide person-centered dementia care at home.

